# *Babesia* Taxa in Sika Deer in Hokkaido, Japan: Prevalence in Questing Ticks and Sympatric Grazing Cattle and Taxonomic Clarification

**DOI:** 10.3390/ani16213532

**Published:** 2026-11-08

**Authors:** Ikumi Yamamoto, Yihong Ma, Rika Umemiya-Shirafuji, Shinya Fukumoto, Thillaiampalam Sivakumar, Naoaki Yokoyama

**Affiliations:** 1 National Research Center for Protozoan Diseases, Obihiro University of Agriculture and Veterinary Medicine, Obihiro 080-8555, Hokkaido, Japan; 2 Laboratory of Parasitology, School of Veterinary Medicine, Rakuno Gakuen University, Ebetsu 069-8501, Hokkaido, Japan

**Keywords:** *Babesia*, cattle, Hokkaido, *Ixodes persulcatus*, sika deer

## Abstract

Cervids harbor several *Babesia* taxa, but their potential to infect cattle remains poorly understood. The present study detected *Babesia* taxa in wild sika deer in Hokkaido, Japan, and subsequently examined questing ticks collected from cattle pastures and sympatric grazing cattle for their presence. The initial screening of 285 sika deer blood DNA samples using a PCR assay targeting the 18S rRNA of *Babesia* spp., followed by sequencing, identified two previously reported *Babesia* taxa: one closely related to *Babesia divergens* and designated *Babesia* sp. Sika 1, and another unclassified *Babesia* taxon designated *Babesia* sp. Sika 2. A duplex PCR assay based on the obtained 18S rRNA sequences was then used to detect these two taxa in 285 sika deer, 671 questing ticks, and 767 cattle. *Babesia* sp. Sika 1 was detected in 86/285 (30.2%) deer and 7/367 (1.9%) *Ixodes persulcatus* ticks, whereas *Babesia* sp. Sika 2 was detected in 4/285 (1.4%) deer but not in the examined ticks. Neither taxon was detected in any cattle samples. Phylogenetic analyses placed *Babesia* sp. Sika 1 in a distinct lineage from *B. divergens*, supporting its recognition as a distinct *Babesia* taxon. Similarly, *Babesia* sp. Sika 2 formed a distinct phylogenetic clade in the 18S rRNA analysis. Despite opportunities for exposure through shared pasture environments and *I. persulcatus* feeding on both hosts, neither taxon was detected in the examined cattle. Further studies are now essential to confirm and extend our findings regarding the taxonomic status and host associations of *Babesia* sp. Sika 1 and *Babesia* sp. Sika 2.

## Simple Summary

Wild sika deer in Hokkaido, Japan, are known to harbor two genetically distinct *Babesia* taxa. One is closely related to *Babesia divergens*, a cattle parasite found in Europe, and was designated *Babesia* sp. Sika 1. The other is an unclassified *Babesia* taxon, designated *Babesia* sp. Sika 2. In Hokkaido, sika deer and grazing cattle increasingly share the same environments, where ticks can feed on both hosts. We therefore investigated whether these *Babesia* taxa can also infect cattle. Both taxa were found in sika deer, and *Babesia* sp. Sika 1 was also found in *Ixodes persulcatus* ticks, but neither taxon was detected in cattle. Genetic analyses further showed that *Babesia* sp. Sika 1 forms a lineage separate from *B. divergens*, supporting its interpretation as a distinct *Babesia* lineage rather than its direct assignment to *B. divergens*. Further studies are essential to clarify the findings of the present study regarding the taxonomic status and host associations of *Babesia* sp. Sika 1 and *Babesia* sp. Sika 2.

## 1. Introduction

Babesiosis is a tick-borne infectious disease caused by protozoan parasites of the genus *Babesia*, which infect a wide range of vertebrate hosts, including domestic and wild animals and humans [1]. These parasites are of veterinary and public health importance worldwide, as the infections can cause clinical manifestations such as fever, anemia, hemoglobinuria, and, in severe cases, death [2,3]. In particular, bovine babesiosis is of major concern because of its clinical impact and economic significance associated with production losses, treatment costs, and implementation of control measures [3].

Of the *Babesia* species infecting cattle, *Babesia bovis*, *Babesia bigemina*, *Babesia divergens*, and *Babesia naoakii* are major causative agents of clinical bovine babesiosis [3,4,5]. Among them, *B. divergens* is distributed in parts of Europe and Africa, corresponding to the geographical distribution of its vector, *Ixodes ricinus* [4]. *Babesia divergens* is also a zoonotic *Babesia* species capable of causing human babesiosis [6]. Interestingly, several *B. divergens*-related parasites have also been reported in cervid hosts [7].

In Japan, previous epidemiological studies identified a *B. divergens*-like parasite in sika deer (*Cervus nippon*) and *Ixodes persulcatus* ticks [8,9,10,11]. Based on 18S rRNA sequences, this parasite forms a lineage closely related to *B. divergens* [9,10]. In addition to the *B. divergens*-like parasite, another unclassified *Babesia* sp. has also been detected in sika deer in Japan [12,13]. Therefore, at least two genetically distinct *Babesia* taxa have been reported in sika deer in Japan. For clarity, the *B. divergens*-like taxon and the previously unclassified *Babesia* taxon identified in sika deer in Japan are provisionally referred to as *Babesia* sp. Sika 1 and *Babesia* sp. Sika 2, respectively, throughout this manuscript.

The taxonomy of *B. divergens* and closely related *Babesia* taxa has historically been difficult to resolve, particularly when based on 18S rRNA sequences alone [14]. Earlier phylogenetic studies placed *B. divergens*, *Babesia capreoli*, *Babesia* sp. MO1, and *Babesia* sp. Sika 1 within a closely related group, making species-level assignment difficult [8]. More recently, *Babesia* sp. MO1 was formally described as *Babesia hegotelforum*, establishing it as a species distinct from *B. divergens* based on genome-wide sequence divergence, phylogenetic placement, host associations, and other biological characteristics [15]. This revised taxonomic framework provides an opportunity to reassess the phylogenetic position of *Babesia* sp. Sika 1.

The expansion of sika deer populations in Japan has increased ecological overlap between wild deer and grazing cattle [16]. In Hokkaido, deer frequently inhabit or move through cattle pastures, increasing opportunities for exposure to the same tick species [17]. Moreover, *I. persulcatus*, a potential vector of *Babesia* taxa infecting sika deer in Hokkaido, is known to feed on both deer and cattle [18,19,20]. Therefore, cattle may encounter ticks carrying these *Babesia* parasites within shared pasture environments. However, such ecological contact does not necessarily result in cattle infection. For example, a previous study in Hokkaido detected a *Theileria* species infecting sika deer in both deer and questing ticks collected from cattle pastures, but not in sympatric grazing cattle [21]. Whether the same pattern applies to *Babesia* taxa detected in sika deer remains unknown, as no epidemiological study has examined their potential to infect sympatric grazing cattle in Japan. Based on this epidemiological context, the present study first identified *Babesia* taxa detected in sika deer in Hokkaido and subsequently surveyed questing ticks collected from cattle pastures and sympatric grazing cattle for these parasites to assess their potential to infect cattle, while reassessing the taxonomic position of *Babesia* sp. Sika 1 using phylogenetic analyses.

## 2. Materials and Methods

### 2.1. DNA Samples from Wild Sika Deer, Grazing Cattle, and Questing Ticks

**Figure 1:**
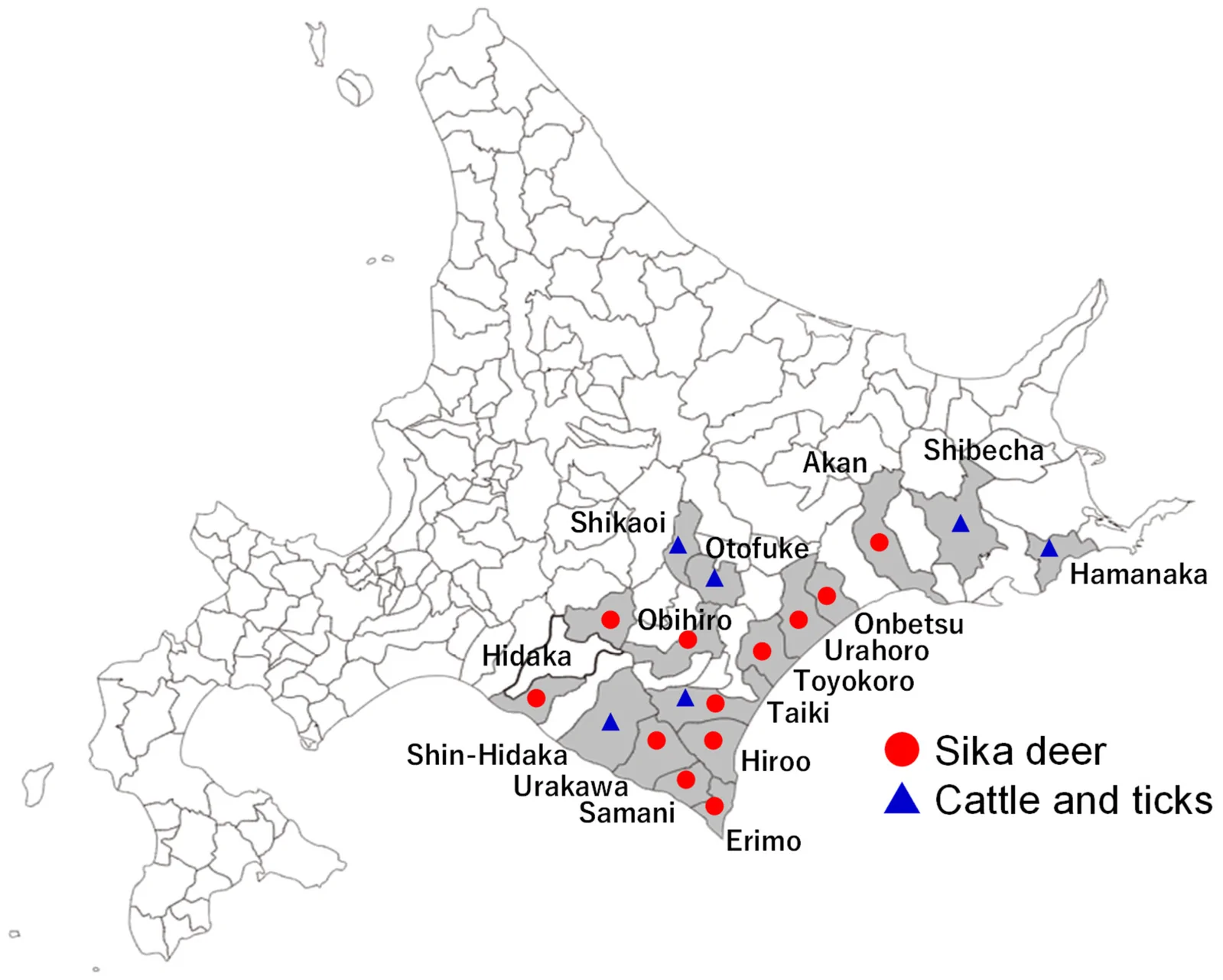
Map showing the sampling locations of sika deer, cattle, and questing ticks in Hokkaido, Japan.

Archived DNA samples from the blood of 285 wild sika deer [21,22] and blood of 767 grazing cattle [21] raised in the same or adjacent regions as the deer sampling sites, and 671 questing ticks collected from cattle pastures [21] in Hokkaido, Japan, were used in the present study. The deer samples were originally obtained from deer during hunting or slaughter at deer farms. Detailed sampling locations and sample numbers for the deer samples are provided in Table 1, and the sampling locations of the deer, cattle, and ticks are shown in Figure 1. Some of these samples were used in previous studies [21,22]. Based on morphological characteristics, the ticks were previously identified as *I. persulcatus*, *Ixodes ovatus*, *Haemaphysalis japonica*, and *Haemaphysalis megaspinosa* (Table 2) [21,23].

The deer and cattle DNA samples were extracted from whole blood using the QIAamp DNA Blood Mini Kit (Qiagen, Hilden, Germany), while tick DNA was extracted using phenol:chloroform:isoamyl alcohol after digestion with the lysis buffer provided in the ISOHAIR kit (Nippon Gene, Tokyo, Japan). All DNA samples were extracted within a short period after sample collection and stored at −30 °C in aliquots to minimize repeated freeze–thaw cycles.

### 2.2. Molecular Identification of Babesia Taxa Infecting Sika Deer

To identify *Babesia* taxa infecting sika deer, all 285 deer DNA samples were screened using a previously described universal PCR assay targeting the 18S rRNA sequences of *Babesia* spp. [24] (forward primer: 5′-CATTACAACAGTTATAGTTTCTTTGG-3′; reverse primer: 5′-CTAGGCATTCCTCGTTCATGATTTAG-3′). Briefly, a 10-μL PCR reaction mixture was prepared containing 2 μL of DNA template, 1.0 μL of 10× PCR buffer (Applied Biosystems, Foster City, CA, USA), 1.0 μL of dNTP mixture (Applied Biosystems), 0.5 μL of forward primer (10 μM), 0.5 μL of reverse primer (10 μM), 0.1 μL of AmpliTaq Gold DNA polymerase (Applied Biosystems), and 4.9 μL of nuclease-free water. The mixture was subjected to the following cycling conditions: initial denaturation at 95 °C for 5 min; 40 cycles of denaturation at 95 °C for 30 s, annealing at 62 °C for 1 min, and extension at 72 °C for 2 min; and a final extension at 72 °C for 10 min. PCR products were analyzed by electrophoresis on 1.5% agarose gels, stained with ethidium bromide, and visualized under UV light. The presence of an approximately 1400-bp amplicon indicated a positive result for *Babesia* spp. Amplicons of the expected size (~1400 bp) were purified, cloned into the pCR2.1-TOPO vector (Invitrogen, Carlsbad, CA, USA), and sequenced. All amplicons obtained from the 28 *Babesia* spp. PCR-positive deer samples were subjected to sequencing.

Sequence analysis identified the two previously described *Babesia* taxa in sika deer in Japan, namely *Babesia* sp. Sika 1 and *Babesia* sp. Sika 2. To further characterize *Babesia* sp. Sika 1, a 1271-bp fragment of the beta-tubulin gene was amplified from selected *Babesia* sp. Sika 1-positive deer samples using *Babesia*-specific forward (5′-AGATCGGTGCCAARTTCTGGGAG-3′) and reverse (5′-TCCGATACCCTCTTGAACATGTC-3′) primers [8]. The PCR reaction mixture and cycling conditions were the same as those described above, except that the annealing temperature was adjusted to 57 °C. Following agarose gel electrophoresis and ethidium bromide staining, the presence of a 1271-bp product under UV light indicated a positive result. The amplicons were cloned and sequenced.

### 2.3. Duplex PCR Assay for Simultaneous Detection of Babesia sp. Sika 1 and Babesia sp. Sika 2

A duplex PCR assay was used for simultaneous detection of *Babesia* sp. Sika 1 and *Babesia* sp. Sika 2. Briefly, the 18S rRNA sequences obtained from positive sika deer samples were aligned with available relevant sequences of hemopathogens infecting animals in Hokkaido, as well as sequences from sika deer, ticks, and cattle. The alignment was used to identify regions specific to *Babesia* sp. Sika 1 and *Babesia* sp. Sika 2. Taxon-specific forward primers for *Babesia* sp. Sika 1 (5′-GGGGAAACTCACCAGGTCCAGACAAT-3′) and *Babesia* sp. Sika 2 (5′-CAGCCTGTGGCATTCTCGCGCGTGC-3′) were then manually designed, with particular attention to sequence differences at or near the 3′ ends of the primers. A common reverse primer (5′-CTAGGCATTCCTCGTTCATGATTTAG-3′) was selected from a region conserved between *Babesia* sp. Sika 1 and *Babesia* sp. Sika 2 but divergent from non-target sequences.

PCR amplification was performed in a final reaction volume of 10 μL containing 2 μL of DNA template, 1.0 μL of 10× PCR buffer (Applied Biosystems, Foster City, CA, USA), 1.0 μL of dNTP mixture (Applied Biosystems), 0.5 μL of each forward primer (10 μM), 0.5 μL of reverse primer (10 μM), 0.1 μL of AmpliTaq Gold DNA polymerase (Applied Biosystems), and 4.4 μL of nuclease-free water. Thermal cycling conditions consisted of an initial denaturation at 95 °C for 5 min, followed by 40 cycles of denaturation at 95 °C for 30 s, annealing at 62 °C for 1 min, and extension at 72 °C for 2 min, with a final extension at 72 °C for 5 min. PCR products were analyzed by electrophoresis on 1.5% agarose gels, stained with ethidium bromide, and observed under UV light. The presence of 394-bp and 889-bp amplicons indicated positive results for *Babesia* sp. Sika 1 and *Babesia* sp. Sika 2, respectively.

The specificity of the duplex PCR assay was evaluated using DNA samples from related hemopathogens, including *B. bovis*, *B. bigemina*, *Babesia ovata*, *B. naoakii*, *Babesia* sp. Hue-1, *Theileria orientalis*, *Theileria annulata*, *Theileria parva*, *Theileria* sp. Sika-1, *Trypanosoma evansi*, *Trypanosoma brucei*, *Trypanosoma theileri*, *Anaplasma marginale*, and *Anaplasma centrale*, as well as uninfected bovine blood DNA [21,24].

### 2.4. Detection of Babesia sp. Sika 1 and Babesia sp. Sika 2 in Deer, Ticks, and Cattle Using Duplex PCR Assay

The duplex PCR assay was used to detect *Babesia* sp. Sika 1 and *Babesia* sp. Sika 2 in DNA samples obtained from wild sika deer, questing ticks, and grazing cattle. Two *I. persulcatus* DNA samples that tested positive using the duplex PCR assay were then subjected to the same universal 18S rRNA PCR assay used for the initial detection of *Babesia* taxa in deer. The resulting amplicons were cloned and sequenced to confirm the duplex PCR results.

### 2.5. Phylogenetic Analysis

Phylogenetic trees were constructed separately for three sequence datasets: 18S rRNA sequences of *Babesia* sp. Sika 1 from sika deer and ticks, beta-tubulin gene sequences of *Babesia* sp. Sika 1 from positive sika deer, and 18S rRNA sequences of *Babesia* sp. Sika 2 from sika deer. Separate 18S rRNA phylogenetic trees were constructed for *Babesia* sp. Sika 1 and *Babesia* sp. Sika 2 as they addressed different phylogenetic questions. For *Babesia* sp. Sika 1, closely related taxa were selected to improve resolution of its relationship with *B. divergens* and other closely related taxa, whereas a broader set of *Babesia* species was included to assess the phylogenetic position of *Babesia* sp. Sika 2.

The sequences obtained in the present study, together with reference sequences retrieved from GenBank, were aligned using MAFFT online software (version 7) [25]. The resulting alignments were analyzed using MEGA version 11 to determine the best-fitting nucleotide substitution models [26]. Maximum likelihood phylogenetic trees were constructed using MEGA11 based on the Tamura–Nei substitution model [27], with the complete deletion option selected to exclude alignment positions containing gaps and missing data. The final numbers of nucleotide positions included in the phylogenetic analyses were 1413 bp for the 18S rRNA sequences of *Babesia* sp. Sika 1; 902 bp for the beta-tubulin sequences of *Babesia* sp. Sika 1; and 1226 bp for the 18S rRNA sequences of *Babesia* sp. Sika 2.

## 3. Results

### 3.1. Babesia Taxa Detected in Wild Sika Deer in Hokkaido

Among 285 blood DNA samples from sika deer, 28 (9.8%) tested positive using the initial universal PCR targeting the 18S rRNA sequences of *Babesia* spp., yielding amplicons of the expected size (~1400 bp). Sequence analysis of the 28 PCR amplicons identified two genetically distinct *Babesia* lineages, which were designated *Babesia* sp. Sika 1 and *Babesia* sp. Sika 2. Of these, 25 samples were positive only for *Babesia* sp. Sika 1, two were positive only for *Babesia* sp. Sika 2, and one was positive for both taxa (Table 1). The obtained sequences (Accession Nos. LC951429-LC951454) were 1420–1423 bp in length and shared 99.5–99.9% sequence identities with previously reported 18S rRNA sequences of *Babesia* sp. Sika 1 (Accession Nos. KC493555, AB975389, LC279018, KC465977, and KC465976).

On the other hand, *Babesia* sp. Sika 2 was detected only in three deer samples, including one sample that was co-infected with *Babesia* sp. Sika 1. The obtained sequences (Accession Nos. LC951455-LC951457) were 1392 bp in length and shared 99.8% sequence identity with previously reported 18S rRNA sequences of *Babesia* sp. Sika 2 from sika deer in Japan (Accession No. LC536169).

**Figure 2:**
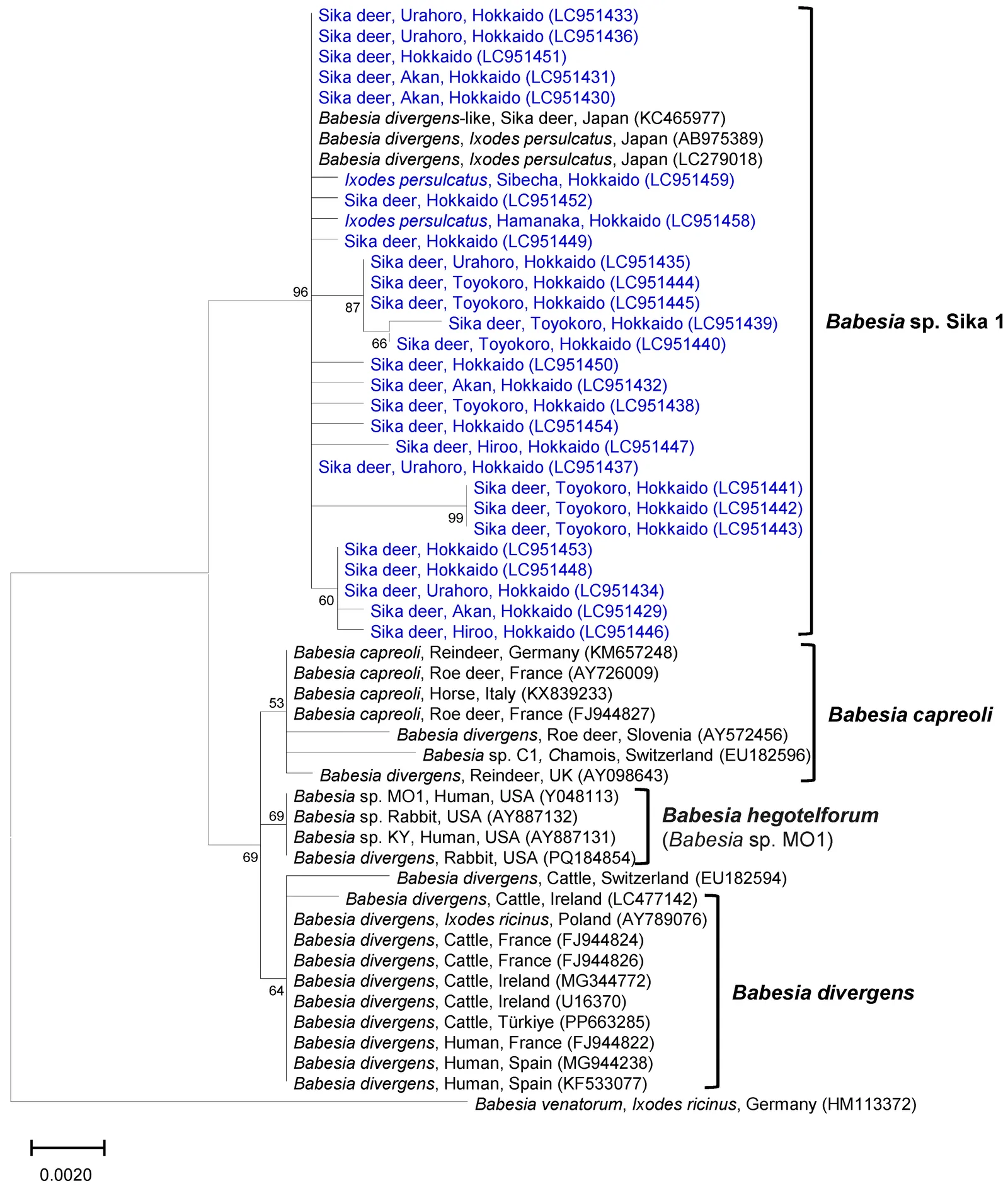
Phylogenetic analysis of 18S rRNA sequences of *Babesia* sp. Sika 1 obtained from sika deer and questing ticks. The sequences obtained in this study (highlighted in blue) clustered with previously reported *Babesia* sp. Sika 1 sequences and were sister to the common ancestor of *Babesia divergens*, *Babesia capreoli*, and *Babesia hegotelforum*.

**Figure 3:**
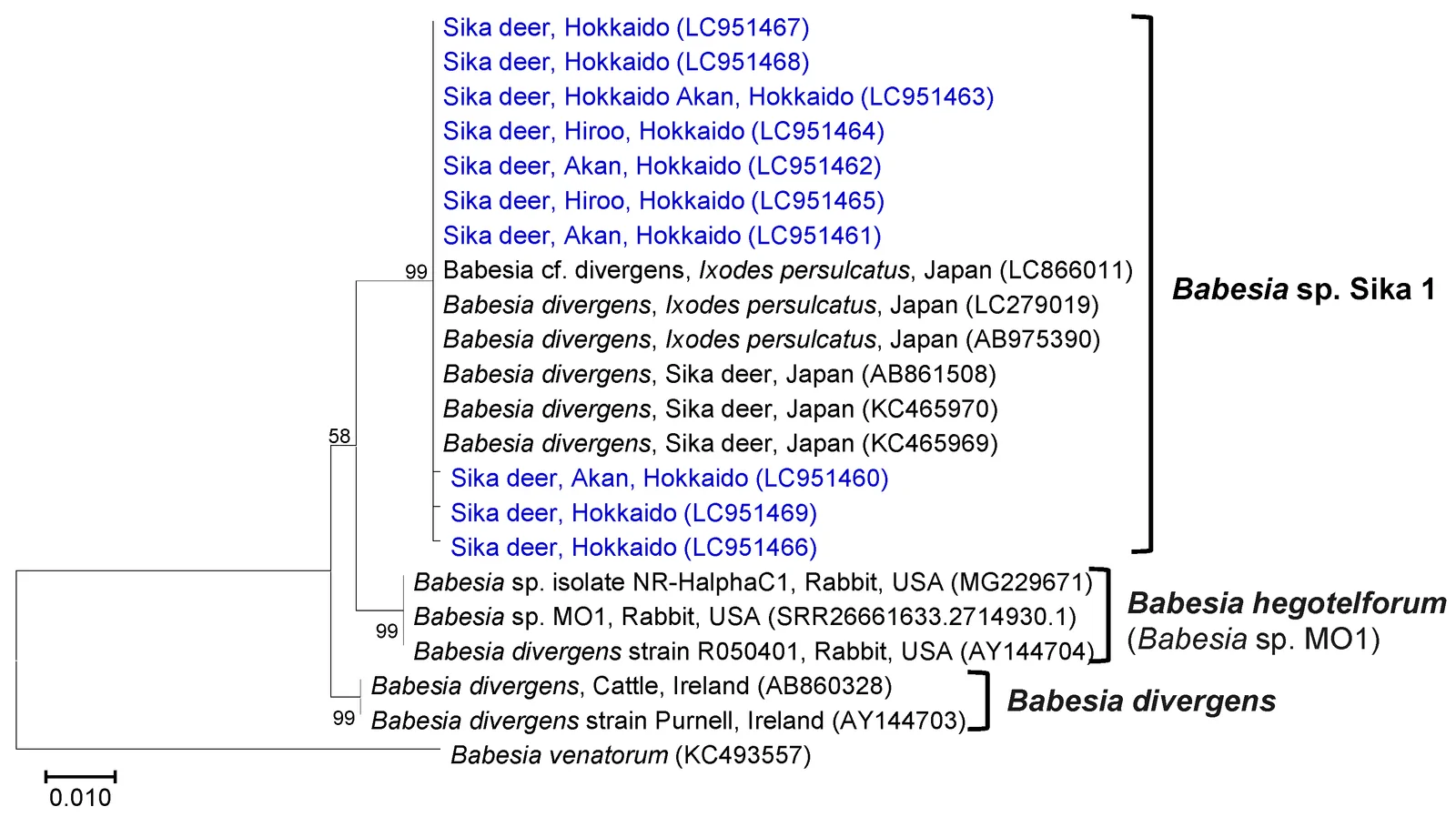
Phylogenetic analysis of beta-tubulin sequences of *Babesia* sp. Sika 1 obtained from sika deer. The sequences obtained in this study (highlighted in blue) clustered with previously reported *Babesia* sp. Sika 1 sequences and were sister to the *Babesia hegotelforum* clade. The common ancestor of these two clades was sister to the *Babesia divergens* clade.

In the 18S rRNA phylogeny, the newly obtained *Babesia* sp. Sika 1 sequences clustered with previously reported *Babesia* sp. Sika 1 sequences from sika deer and *I. persulcatus* ticks in Japan (Figure 2). The *Babesia* sp. Sika 1 clade was sister to the common ancestor of *B. divergens*, *B. capreoli*, and *B*. *hegotelforum* (formerly *Babesia* sp. MO1). The analysis of beta-tubulin gene sequences (Accession Nos. LC951460-LC951469) from *Babesia* sp. Sika 1-positive deer further supported the distinct phylogenetic position of *Babesia* sp. Sika 1 relative to *B. divergens* (Figure 3). The obtained sequences formed a sister clade to *B. hegotelforum*, and the common ancestor of these two clades was sister to the *B. divergens* clade (Figure 3).

**Figure 4:**
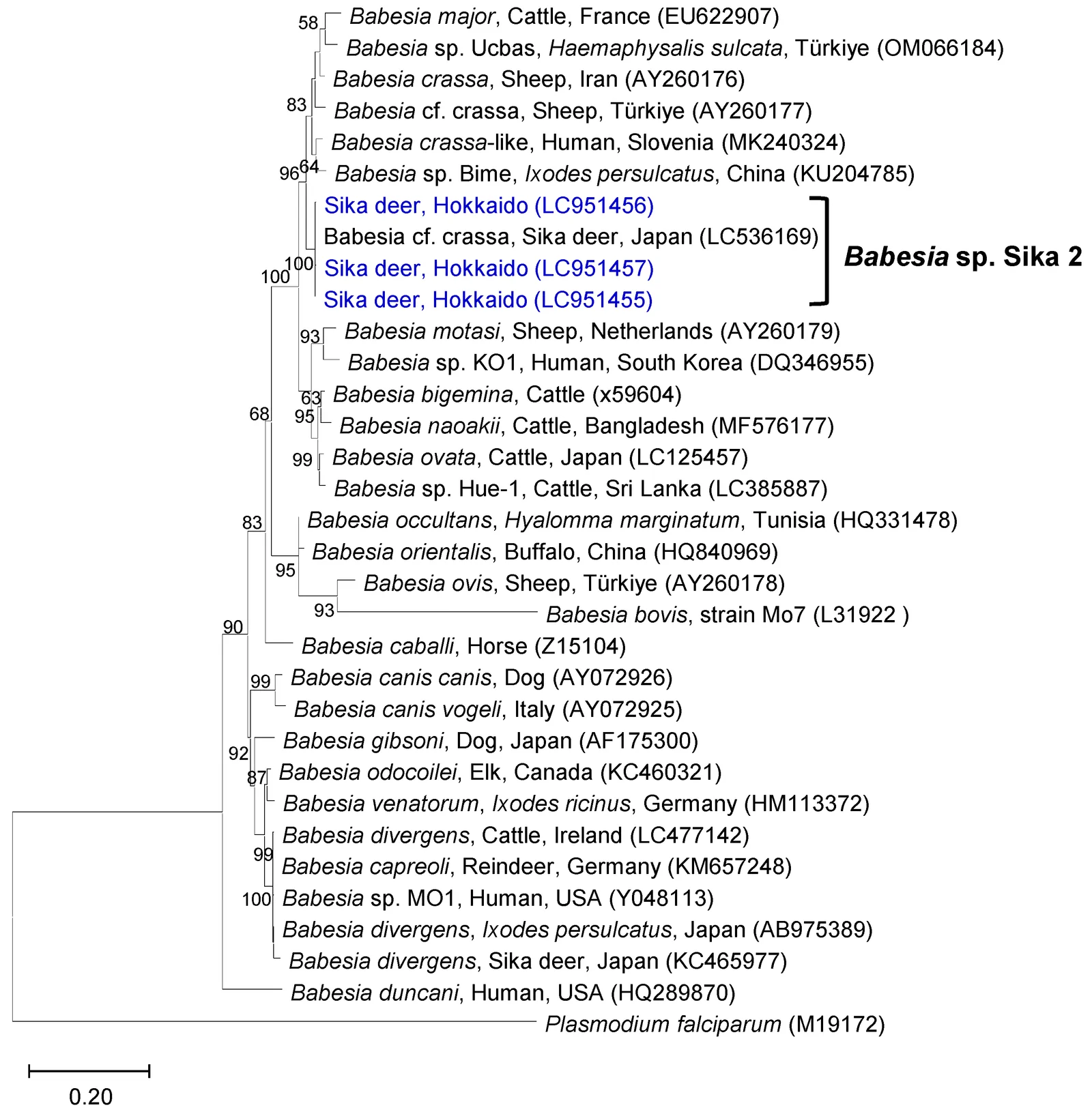
Phylogenetic analysis of 18S rRNA sequences of *Babesia* sp. Sika 2 obtained from sika deer. The sequences obtained in this study (highlighted in blue) clustered with a previously reported *Babesia* sp. Sika 2 sequence and were sister to the common ancestor of *Babesia crassa*, *Babesia crassa*-like species, and *Babesia major*.

In the 18S rRNA phylogeny, *Babesia* sp. Sika 2 sequences clustered with a previously reported *Babesia* sequence from sika deer in Japan, and formed a sister clade to the common ancestor of *Babesia major*, *Babesia crassa*, and *B. crassa*-like species (Figure 4).

### 3.2. Distribution of Babesia sp. Sika 1 and Babesia sp. Sika 2 in Deer, Ticks, and Cattle as Determined by Duplex PCR Assay

Based on the sequence polymorphisms in 18S rRNA, taxon-specific primers were designed for simultaneous detection of *Babesia* sp. Sika 1 and *Babesia* sp. Sika 2 by duplex PCR assay. The assay produced the expected taxon-specific amplicons, but no amplification was observed from the other tested hemoparasites and uninfected bovine blood DNA, indicating specificity for the tested DNA samples.

The duplex PCR assay was then used to screen all the deer, tick, and cattle DNA samples. *Babesia* sp. Sika 1 was detected in 86/285 deer samples (30.2%), with positive deer distributed across multiple sampling sites in Hokkaido (Table 1). By contrast, *Babesia* sp. Sika 2 was detected in 4/285 deer samples (1.4%) at only a limited number of sampling sites. All samples identified as positive for *Babesia* sp. Sika 1 or *Babesia* sp. Sika 2 by the initial universal PCR and sequencing were also detected as positive for the corresponding taxon by duplex PCR. The sample identified as co-infected with both taxa by the initial PCR and sequencing was also positive for both taxa using the duplex PCR. Among the samples identified as negative by the initial PCR, the duplex PCR additionally detected *Babesia* sp. Sika 1 in 60 samples and *Babesia* sp. Sika 2 in one sample; no additional co-infections were detected.

Among the 671 tick DNA samples, *Babesia* sp. Sika 1 was detected in 7/367 (1.9%) *I. persulcatus* ticks collected from Hamanaka, Shibecha, and Taiki, while no other tick species tested positive for this taxon (Table 2). Positive ticks included both adults and nymphs, whereas no other tick species tested positive. By contrast, *Babesia* sp. Sika 2 was not detected in any tick samples. The *Babesia* sp. Sika 1-positive ticks included both adults and nymphs. Two selected *I. persulcatus* DNA samples that tested positive for *Babesia* sp. Sika 1 were additionally subjected to the universal 18S rRNA PCR assay. The sequences (Accession Nos. LC951458-LC951459) obtained from the resulting amplicons shared 99.2–99.9% sequence identity with the deer-derived *Babesia* sp. Sika 1 sequences and clustered with these sequences in the phylogenetic analysis, supporting the duplex PCR results (Figure 2).

Blood DNA samples from a total of 767 cattle from six districts in Hokkaido, including 50, 100, 300, 77, 146, and 94 cattle from Hamanaka, Shibecha, Shikaoi, Otofuke, Taiki, and Shin-Hidaka districts, respectively, that had been collected between 2008 and 2016 [21], were screened using the duplex PCR assay. We found that none of the 767 DNA samples tested positive for either *Babesia* sp. Sika 1 or *Babesia* sp. Sika 2.

## 4. Discussion

The present study identified two genetically distinct *Babesia* lineages in sika deer in Hokkaido prefecture and designated them *Babesia* sp. Sika 1 and *Babesia* sp. Sika 2. Of these two taxa, *Babesia* sp. Sika 1 was also detected in *I. persulcatus* ticks collected from cattle pastures, whereas neither taxon was detected in the examined cattle. These findings provided an opportunity to examine whether *Babesia* taxa infecting sika deer, particularly *Babesia* sp. Sika 1, which is closely related to the bovine parasite *B. divergens*, can infect cattle when opportunities for transmission are present within the deer–tick–cattle interface.

### 4.1. Taxonomic Clarification and Host and Tick Associations of Babesia sp. Sika 1

A relatively high proportion of the sika deer examined in the present study were identified as positive for *Babesia* sp. Sika 1 by the taxon-specific duplex PCR assay. The detection rate obtained with the duplex PCR was substantially higher than that obtained with the initial *Babesia* spp. PCR. This difference may reflect differences in amplification efficiency between the two assays. The initial PCR used broad-range primers targeting a relatively long 18S rRNA fragment, whereas the duplex PCR used a *Babesia* sp. Sika 1-specific forward primer targeting a shorter region. In addition, the primers used for the initial PCR may not completely match the 18S rRNA sequence of *Babesia* sp. Sika 1, which may have reduced amplification efficiency. These differences may have contributed to the higher detection rate of *Babesia* sp. Sika 1 obtained with the duplex PCR assay.

In the present study, the 18S rRNA phylogeny separated *Babesia* sp. Sika 1 from *B. divergens*, *B. capreoli*, and *B. hegotelforum*, placing it in a distinct lineage. Analysis of beta-tubulin sequences also placed *Babesia* sp. Sika 1 separately from *B. divergens*, although the topology differed from that obtained with 18S rRNA sequences. The concordant separation across two independent molecular markers strengthens the evidence that *Babesia* sp. Sika 1 represents a distinct *Babesia* lineage, although these phylogenetic findings do not by themselves establish formal species status. Some sequences derived from hosts other than cattle and humans that were previously identified as *B. divergens* were also positioned outside the *B. divergens* clade. Their phylogenetic positions and reported host origins suggest that some of these sequences may represent other *Babesia* taxa rather than *B. divergens*. Together, these phylogenetic findings support the recognition of *Babesia* sp. Sika 1 as a distinct *Babesia* taxon rather than direct assignment to *B. divergens*.

The phylogenetically distinct *Babesia* species closely related to *B. divergens* also differs in their vertebrate hosts and *Ixodes* tick species. *Babesia divergens* primarily infects cattle and is transmitted by *I. ricinus* in Europe [4], whereas *B. capreoli* mainly infects cervids in Europe and is transmitted by *I. ricinus* [28], and *B. hegotelforum* has been reported from cottontail rabbits and is transmitted by *Ixodes dentatus* in North America [15]. In addition, *B. divergens* and *B. hegotelforum* are zoonotic species capable of infecting humans [4,29]. In contrast, *Babesia* sp. Sika 1 has been frequently detected in sika deer and was also detected in *I. persulcatus* in Hokkaido, consistent with previous reports of this parasite in the same deer–tick system [8,9,11]. Therefore, the distinct phylogenetic position, together with differences in vertebrate hosts and tick vectors compared with *B. divergens*, further supports the recognition of *Babesia* sp. Sika 1 as a distinct *Babesia* lineage.

Accurate taxonomic recognition of *Babesia* sp. Sika 1 is important because referring to this parasite as *B. divergens*-like or as the *B. divergens* Asian lineage may lead to assumptions about its host range and epidemiological significance based on the established biology of *B. divergens*. The present study provided phylogenetic evidence that *Babesia* sp. Sika 1 is distinct from *B. divergens*, but the available data are not sufficient for formal species description. Formal taxonomic recognition would require additional biological and genomic evidence, including morphological characterization and comparative genomic analysis with *B. divergens* and other closely related taxa [15]. Such information would provide the additional evidence needed to formally describe *Babesia* sp. Sika 1 as a separate *Babesia* species.

### 4.2. Babesia sp. Sika 2 in Sika Deer, Ticks, and Cattle

Unlike *Babesia* sp. Sika 1, *Babesia* sp. Sika 2 has been poorly characterized, with only limited information available on its distribution and host associations [13]. In the 18S rRNA phylogeny, *Babesia* sp. Sika 2 formed a distinct phylogenetic clade separate from the other *Babesia* species included in the analysis. However, its biological and epidemiological characteristics remain largely unknown. In contrast to *Babesia* sp. Sika 1, the prevalence of *Babesia* sp. Sika 2 was very low in the wild sika deer population, and this taxon was not detected in any of the questing ticks. These contrasting patterns of the two taxa suggest that genetically distinct *Babesia* taxa may differ substantially in their prevalence within the same wildlife host population and their distribution among questing tick populations.

The low prevalence of *Babesia* sp. Sika 2 in deer may be related to low parasite burdens, which could reduce the probability of detection by PCR [30]. Alternatively, it is possible that other vertebrate hosts may serve as the primary hosts for *Babesia* sp. Sika 2, while sika deer may represent a spillover host rather than the principal host [31]. The low prevalence of *Babesia* sp. Sika 2 in the deer examined in the present study may have limited the opportunities for acquisition by feeding ticks and consequently contributed to its absence from the tick samples. Alternatively, *Babesia* sp. Sika 2 may be transmitted by tick species other than those surveyed in the present study. Further studies involving additional tick species, other sympatric wildlife species, and broader geographic sampling would help clarify the host range and transmission ecology of *Babesia* sp. Sika 2.

### 4.3. Epidemiological Implications at the Deer–Tick–Cattle Interface

The present study provided field evidence on *Babesia* taxa at the deer–tick–cattle interface in Hokkaido. Wild sika deer and grazing cattle share pasture environments, and *I. persulcatus* feeds on both hosts [18,19,20]. Despite this ecological overlap, neither of the two *Babesia* taxa examined in the present study was detected in cattle. A similar pattern has been reported for a *Theileria* species in Hokkaido, which was detected in sika deer and questing ticks collected from cattle pastures, but not in sympatric grazing cattle [21]. Together, these observations suggest that the presence of tick-borne protozoan parasites in wildlife and questing ticks within cattle environments may not necessarily result in detectable infection of cattle.

The absence of *Babesia* sp. Sika 1 in cattle is notable given its close phylogenetic relationship with *B. divergens*, which primarily infects cattle [4]. The present field observations suggest that close phylogenetic relatedness does not necessarily correspond to a similar host range. Host specificity may instead depend on biological compatibility between the parasite and its vertebrate host.

Biological compatibility may depend on the ability of the parasites to establish and multiply within host cells. In *Babesia*, infection involves a successful invasion of erythrocytes followed by intracellular development and multiplication, and differences in these processes may contribute to host range [32,33]. For example, an in vitro study of *B. bovis* showed that host specificity can involve differences in erythrocyte invasion, multiplication, and reinvasion among host species [34]. Similar differences in erythrocyte invasion or intracellular development could contribute to the absence of *Babesia* sp. Sika 1 from cattle. Therefore, additional studies are needed to experimentally examine the interaction of *Babesia* sp. Sika 1 with bovine erythrocytes and determine whether its absence from cattle reflects limited compatibility with bovine erythrocytes.

Successful isolation and in vitro cultivation of *Babesia* sp. Sika 1 would enable comparative experiments using sika deer and bovine erythrocytes to directly examine erythrocyte invasion, intracellular development, and multiplication. In parallel, larger-scale epidemiological studies using deer, questing ticks, and sympatric cattle collected from multiple geographic regions would help determine whether the absence of *Babesia* sp. Sika 1 and *Babesia* sp. Sika 2 from cattle is consistent across different ecological settings. These approaches could clarify the host range and transmission ecology of *Babesia* taxa detected in sika deer in Hokkaido, Japan.

### 4.4. Limitations of the Study

Several limitations should be considered when interpreting the present findings. First, the analytical sensitivity of the duplex PCR assay was not formally evaluated, and its ability to detect low levels of *Babesia* sp. Sika 1 and *Babesia* sp. Sika 2 is therefore unknown. This limitation is particularly relevant to the low detection rate of *Babesia* sp. Sika 2 and to the interpretation of negative results. In addition, preferential amplification of *Babesia* sp. Sika 1 over *Babesia* sp. Sika 2 in samples containing both taxa cannot be excluded.

Second, the molecular evidence available for the two taxa was not equivalent. *Babesia* sp. Sika 1 was characterized using both 18S rRNA and beta-tubulin sequences, whereas *Babesia* sp. Sika 2 was characterized only by 18S rRNA sequences. Therefore, the phylogenetic evidence supporting the distinct position of *Babesia* sp. Sika 2 is more limited, and additional genetic markers will be needed to clarify its taxonomic position.

Third, the deer, tick, and cattle samples were collected during different sampling periods. Although these hosts share environments and *I. persulcatus* feeds on both deer and cattle, the present study therefore does not demonstrate contemporaneous circulation or direct transmission of the same *Babesia* taxa among these hosts.

Finally, the absence of detectable *Babesia* sp. Sika 1 and *Babesia* sp. Sika 2 in the examined cattle should be interpreted cautiously. Negative PCR results may reflect low parasite burdens, differences in sample timing, or limitations in assay sensitivity, and the present findings do not establish whether either taxon is biologically incapable of infecting cattle. Larger studies using samples collected contemporaneously from deer, ticks, and cattle, together with more sensitive and analytically validated molecular assays, will be needed to further clarify the transmission potential and host range of these *Babesia* taxa.

## 5. Conclusions

The present study identified two genetically distinct *Babesia* lineages in sika deer in Hokkaido, provisionally designated *Babesia* sp. Sika 1 and *Babesia* sp. Sika 2. While *Babesia* sp. Sika 1 was also detected in questing *I. persulcatus* ticks, neither lineage was detected in the examined grazing cattle. Phylogenetic analyses using 18S rRNA and beta-tubulin sequences supported the distinct position of *Babesia* sp. Sika 1 relative to *B. divergens*, while *Babesia* sp. Sika 2, which was characterized only by 18S rRNA, formed a distinct phylogenetic clade. These findings support the recognition of *Babesia* sp. Sika 1 and *Babesia* sp. Sika 2 as distinct *Babesia* lineages, but do not establish formal species status. The absence of detectable infection in cattle should be interpreted cautiously in view of the limited analytical assessment of the duplex PCR and the different sampling periods among the examined host animals.

## Figures and Tables

**Table 1:** Detection of *Babesia* sp. Sika 1 and *Babesia* sp. Sika 2 in blood DNA samples from sika deer by universal PCR and taxon-specific duplex PCR assays targeting the 18S rRNA.

District	Sampling Period	No. Sample	No. Positive (%)	No. Positive (%) by Duplex PCR	
	(Year/Month)		by Universal PCR	*Babesia* sp. Sika 1	*Babesia* sp. Sika 2
Akan	2011/6–2011/7	51	4 (7.8)	10 (19.6)	0
Onbetsu	2010/12–2011/1	3	0 (0.0)	1 (33.3)	0
Urahoro	2010/12–2012/1	56	6 (10.7)	26 (46.4)	0
Toyokoro	2010/12–2012/2	97	8 (8.2)	26 (26.8)	3 (3.1)
Obihiro	2011/1–2011/12	6	0 (0.0)	2 (33.3)	0
Hidaka	2011/11	2	0 (0.0)	1 (50)	0
Taiki	2011/11	1	0 (0.0)	1 (100)	0
Hiroo	2010/12–2011/12	14	1 (7.1)	4 (28.6)	0
Erimo	2010/9–2011/1	10	0 (0.0)	4 (40)	0
Samani	2010/12	3	0 (0.0)	0	0
Urakawa	2010/12	1	0 (0.0)	0	0
Unknown	2010/5–2013/3	41	9 (22.0)	11 (26.8)	1 (2.4)
Total		285	28 (9.8)	86 (30.2)	4 (1.4)

**Table 2:** Detection of *Babesia* sp. Sika 1 in questing ticks by duplex PCR assay.

Location	Period	*Ixodes persulcatus*		*Ixodes ovatus*		*Haemaphysalis japonica*		*Haemaphysalis megaspinosa*	
	(Year/Month)	No. Sample	Positive (%)	No. Sample	Positive (%)	No. Sample	Positive (%)	No. Sample	Positive (%)
Hamanaka	2015/5&2016/5	23	2 (8.7)	1	0	6	0	0	-
Shibecha	2016/6	80	4 (5.0)	101	0	50	0	0	-
Shikaoi	2016/5&2017/6	56	0	38	0	20	0	2	0
Otofuke	2008/4	41	0	0	-	1	0	0	-
Taiki	2008/4	161	1 (0.6)	5	0	22	0	0	-
Shin-Hidaka	2008/6	6	0	5	0	4	0	49	0
Total		367	7 (1.9)	150	0	103	0	51	0

*Babesia* sp. Sika 1 was detected only in *I. persulcatus* ticks, whereas all surveyed ticks were negative for *Babesia* sp. Sika 2.

## Data Availability

All data generated in the present study are reported in this article.

## References

[1] Homer M.J., Aguilar-Delfin I., Telford S.R., Krause P.J., Persing D.H. (2000). Babesiosis. Clin. Microbiol. Rev..

[2] Vannier E., Gewurz B.E., Krause P.J. (2008). Human babesiosis. Infect. Dis. Clin. N. Am..

[3] Bock R., Jackson L., de Vos A., Jorgensen W. (2004). Babesiosis of cattle. Parasitology.

[4] Zintl A., Mulcahy G., Skerrett H.E., Taylor S.M., Gray J.S. (2003). *Babesia divergens*, a bovine blood parasite of veterinary and zoonotic importance. Clin. Microbiol. Rev..

[5] Sivakumar T., Tuvshintulga B., Otgonsuren D., Batmagnai E., Ahedor B., Kothalawala H., Vimalakumar S.C., Silva S.S.P., Yamagishi J., Yokoyama N. (2022). Phylogenetic analyses of the mitochondrial, plastid, and nuclear genes of *Babesia* sp. Mymensingh and its naming as *Babesia naoakii* n. sp.. Parasites Vectors.

[6] Ord R.L., Lobo C.A. (2015). Human babesiosis: Pathogens, prevalence, diagnosis and treatment. Curr. Clin. Microbiol. Rep..

[7] Fanelli A. (2021). A historical review of *Babesia* spp. associated with deer in Europe: *Babesia divergens*/*Babesia divergens*-like, *Babesia capreoli*, *Babesia venatorum*, *Babesia* cf. *odocoilei*. Vet. Parasitol..

[8] Ma Y., Sivakumar T., Mumbi N.N.M., Umemiya-Shirafuji R., Yokoyama N. (2026). An epidemiological survey of zoonotic *Babesia* species in questing ticks in Hokkaido, Japan, using a newly developed PCR-sequencing approach. Parasitol. Int..

[9] Zamoto-Niikura A., Tsuji M., Imaoka K., Kimura M., Morikawa S., Holman P.J., Hirata H., Ishihara C. (2014). Sika deer carrying *Babesia* parasites closely related to *B. divergens*, Japan. Emerg. Infect. Dis..

[10] Zamoto-Niikura A., Tsuji M., Qiang W., Morikawa S., Hanaki K.I., Holman P.J., Ishihara C. (2018). The *Babesia divergens* Asia lineage is maintained through enzootic cycles between *Ixodes persulcatus* and sika deer in Hokkaido, Japan. Appl. Environ. Microbiol..

[11] Zamoto-Niikura A., Hagiwara K., Imaoka K., Morikawa S., Ishihara C., Hanaki K.I. (2020). Epidemiological survey of *Babesia divergens* Asia lineage in wild sika deer (*Cervus nippon*) by using direct PCR in Japan. Jpn. J. Infect. Dis..

[12] Elbaz E., Moustafa M.A.M., Lee K., Ching A.L.C., Shimozuru M., Sashika M., Nakao R., El-Khodery S.A., Tsubota T. (2020). Utilizing attached hard ticks as pointers to the risk of infection by *Babesia* and *Theileria* species in sika deer (*Cervus nippon yesoensis*), in Japan. Exp. Appl. Acarol..

[13] Masatani T., Hayashi K., Morikawa M., Ozawa M., Kojima I., Okajima M., Takano A., Shimoda H., Maeda K., Matsuu A. (2020). Molecular detection of tick-borne protozoan parasites in sika deer (*Cervus nippon*) from western regions of Japan. Parasitol. Int..

[14] Hrazdilová K., Rybářová M., Široký P., Votýpka J., Zintl A., Burgess H., Steinbauer V., Žákovčík V., Modrý D. (2020). Diversity of *Babesia* spp. in cervid ungulates based on the 18S rDNA and cytochrome c oxidase subunit I phylogenies. Infect. Genet. Evol..

[15] Singh P., Estrada K., Gonzalez L.M., Grande R., Sánchez-Prieto S., Cornillot E., Harb O., Sanchez-Flores A., Montero E., Le Roch K.G. (2026). *Babesia hegotelforum* sp. nov., a zoonotic *Babesia* species previously referred to as *Babesia* sp. MO1. Emerg. Microbes Infect..

[16] Hata A., Tsukada H., Washida A., Mitsunaga T., Takada M.B., Suyama T., Takeuchi M. (2019). Temporal and spatial variation in the risk of grazing damage to sown grasslands by sika deer (*Cervus nippon*) in a mountainous area, central Japan. Crop Prot..

[17] Takafumi H., Kamii T., Murai T., Yoshida R., Sato A., Tachiki Y., Akamatsu R., Yoshida T. (2017). Seasonal and year-round use of the Kushiro Wetland, Hokkaido, Japan by sika deer (*Cervus nippon yesoensis*). PeerJ.

[18] Shimizu K., Shimozuru M., Yamanaka M., Ito G., Nakao R., Tsubota T. (2024). Seasonal infestation patterns of ticks on Hokkaido sika deer (*Cervus nippon yesoensis*). Parasitology.

[19] Shimizu K., Shimozuru M., Tsuruga H., Shirane Y., Yamanaka M., Ito G., Shinjo K., Murakami T., Ijuin A., Kovba A. (2025). Patterns of tick infestation and tick-borne pathogen prevalence in sika deer and brown bears in Hokkaido, Japan. Exp. Appl. Acarol..

[20] Yamane I., Nishiguchi A., Kobayashi S., Zeniya Y. (2006). Cross-sectional survey of ixodid tick species on grazing cattle in Japan. Exp. Appl. Acarol..

[21] Shibata S., Sivakumar T., Igarashi I., Umemiya-Shirafuji R., Inokuma H., Fukumoto S., Yokoyama N. (2018). Epidemiological survey of a cervine *Theileria* in wild deer, questing ticks, and cattle in Hokkaido, Japan. Ticks Tick-Borne Dis..

[22] Shirozu T., Badolo A., Soga A., Yoshimura A., Morishita Y.-K., Koketsu M., Umemiya-Shirafuji R., Inokuma H., Yokoyama N., Fukumoto S. (2020). Development and evaluation of a novel loop-mediated isothermal amplification (LAMP) method targeting *Theileria* parasites infecting Yezo sika deer. Parasitol. Int..

[23] Yamaguti N., Tipton V.J., Keegan H.L., Toshioka S. (1971). Ticks of Japan, Korea, and the Ryukyu Islands. Brigham Young Univ. Sci. Bull. Biol. Ser..

[24] Sivakumar T., Tuvshintulga B., Zhyldyz A., Kothalawala H., Yapa P.R., Kanagaratnam R., Vimalakumar S.C., Abeysekera T.S., Weerasingha A.S., Yamagishi J. (2018). Genetic analysis of *Babesia* isolates from cattle with clinical babesiosis in Sri Lanka. J. Clin. Microbiol..

[25] Katoh K., Rozewicki J., Yamada K.D. (2019). MAFFT online service: Multiple sequence alignment, interactive sequence choice and visualization. Brief. Bioinform..

[26] Tamura K., Stecher G., Kumar S. (2021). MEGA11: Molecular Evolutionary Genetics Analysis version 11. Mol. Biol. Evol..

[27] Tamura K., Nei M. (1993). Estimation of the number of nucleotide substitutions in the control region of mitochondrial DNA in humans and chimpanzees. Mol. Biol. Evol..

[28] Malandrin L., Jouglin M., Sun Y., Brisseau N., Chauvin A. (2010). Redescription of *Babesia* capreoli (Enigk and Friedhoff, 1962) from roe deer (*Capreolus capreolus*): Isolation, *cultivation*, host specificity, molecular characterisation and differentiation from *Babesia divergens*. Int. J. Parasitol..

[29] Montero E., Gray J., Lobo C.A., González L.M. (2022). Babesia and human babesiosis. Pathogens.

[30] Bloch E.M., Lee T.H., Krause P.J., Telford S.R., Montalvo L., Chafets D., Usmani-Brown S., Lepore T.J., Busch M.P. (2013). Development of a real-time polymerase chain reaction assay for sensitive detection and quantitation of *Babesia microti* infection. Transfusion.

[31] Lempereur L., Wirtgen M., Nahayo A., Caron Y., Shiels B., Saegerman C., Losson B., Linden A. (2012). Wild cervids are host for tick vectors of *Babesia* species with zoonotic capability in Belgium. Vector-Borne Zoonotic Dis..

[32] Elsworth B., Duraisingh M.T. (2021). A framework for signaling throughout the life cycle of *Babesia* species. Mol. Microbiol..

[33] Yokoyama N., Okamura M., Igarashi I. (2006). Erythrocyte invasion by *Babesia* parasites: Current advances in the elucidation of the molecular interactions between the protozoan ligands and host receptors in the invasion stage. Vet. Parasitol..

[34] Gaffar F.R., Franssen F.F., de Vries E. (2003). *Babesia bovis* merozoites invade human, ovine, equine, porcine and caprine erythrocytes by a sialic acid-dependent mechanism followed by developmental arrest after a single round of cell fission. Int. J. Parasitol..

